# The Power of Plagues, Second Edition

**DOI:** 10.3201/eid2405.171918

**Published:** 2018-05

**Authors:** Thomas J. Marrie

**Affiliations:** Dalhousie University, Halifax, Nova Scotia, Canada

**Keywords:** plague, pestilence, microorganisms, technology, bacteria

A usual dictionary definition of plague (a highly infectious, usually fatal epidemic disease; a pestilence; https://www.thefreedictionary.com) differs from that used by Irwin W. Sherman in his book The Power of Plagues (Figure). To accomplish his purpose in writing this book (“to make the science of epidemic diseases—plagues—accessible and understandable”), Sherman borrows his definition from historian Asa Briggs: “Plagues are a dramatic unfolding of events; they are stories of discovery, reaction, conflict and resilience of local and administrative structures.”

**Figure:**
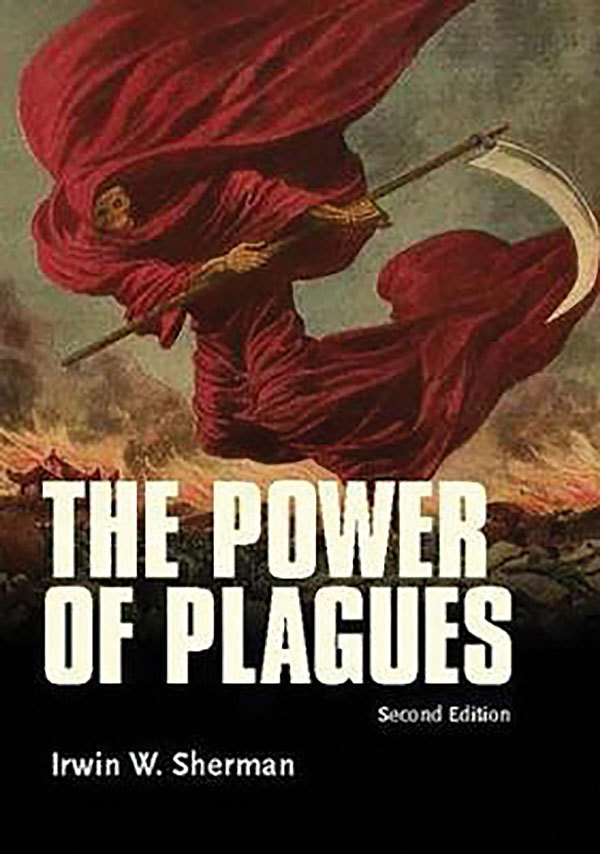
The Power of Plagues

This book is a history of humanity as influenced and shaped by plagues of known and unknown etiology. One of its strengths is also one of its weaknesses. To trace our journey from 4 million years ago to the present, weaving in plagues, people, microorganisms, and advances in technology, is no small feat. However, for the most part, Sherman accomplishes this goal in what is a very readable book that should appeal to a wide variety of audiences. Indeed, it should be read by every student of medicine and the health professions.

Did you know that Pharaoh’s plague and snail fever are the same disease, or that war fever and jail fever are also caused by the same microorganism? Can you name 10 famous people who had syphilis or 20 famous people who died of tuberculosis? Did you know that heroin at one time was a treatment for the very troublesome cough of tuberculosis? Along with the answers to these questions, in this book you will find how plagues shaped history from ancient times to Napoleon’s invasion of Russia to the very modern plagues of HIV/AIDS, influenza, and Lyme disease.

The art or photographic reproductions, usually placed at the beginning of a chapter, are a most powerful method of connecting the reader to what life was like at a given time in history. For example, look at Figure 6.1, Eugen Le Roux’s engraving of Napoleon’s troops in Vilna after the Russian Campaign in 1812, or Figure 5.1, a photograph of Lorraine, age 11, who has AIDS, being comforted by her grandmother.

Errors in the first edition, noted by Rigau-Perez (*1*), have been corrected. The placement of AIDS in Chapter 5 (A 21st Century Plague, AIDS), immediately after the chapter on the Black Death, is out of order chronologically and disrupts the history timeline. Chapters 10 (Preventing Plagues: Immunization) and 11 (The Plague Protectors: Antisepsis to Antibiotics) could easily be deleted in favor of more detail or artwork. However, these criticisms are minor. The major strength of this book is that it is a very readable history of humanity as shaped by plagues, making it attractive to a wide audience.
